# To the Editors of the Medical and Physical Journal

**Published:** 1800-05

**Authors:** John Alderson

**Affiliations:** Hull


### *To the Editors of the Medical and Phyſical Journal.*

GENTLEMEN,

IF you ſhould think the following Luſus Naturæ as ſingular as I do, you will probably give it a place in your uſeful Journal. I am, GENTLEMEN,

Your obedient humble ſervant,

*Hull, April* 9, 1800. JOHN ALDERSON.

---

MRS. ——— informed me ſhe had not been able to give ſuck to her three laſt children, becauſe, when the draught came into her breaſts, the milk flowed out under her arms. On examination I found the mammæ ſmall, and under the fold or lamella of the pectoral muſcle on each ſide, in the fore part of the axilla, a large expanded gland, or ſmall mammæ, perfectly well defined, with a head or nipple like a ſmall wart, formed by the tubi lactiferi; three of which were very diſtinctly obſervable upon preſſure, producing a ſtream of pure milk the ſize of a crow quill.

Theſe glands uniformly became tumid, and filled at the ſame time with the mammæ, and as uniformly poured out the ſecreted fluid before the breaſt ſuffered any conſiderable diſtenſion.

Whenever the child was put to the breaſt, the milk ran in a conſtant ſtream from theſe glands.

She did not obſerve this peculiar conformation till after her ſecond lying-in.

---

### *To the Editors of the Medical and Phyſical Journal.*

GENTLEMEN,

